# Systematic review and meta-analysis of artificial intelligence models for strabismus screening: methodological insights and future directions

**DOI:** 10.1097/JS9.0000000000002916

**Published:** 2025-07-15

**Authors:** Zeyi Yang, Dawen Wu, Jingwen Li, Xiaohang Chen, Longqian Liu

**Affiliations:** a Department of Ophthalmology, West China Hospital, Sichuan University, Chengdu, China; b Department of Optometry and Visual Science, West China Hospital, Sichuan University, Chengdu, China; c West China School of Medicine, Sichuan University, Chengdu, Sichuan, China

**Keywords:** artificial intelligence, screening, strabismus, systematic review and meta-analysis

## Abstract

**Background:**

An increasing number of studies apply artificial intelligence (AI) techniques to strabismus detection to support clinical diagnosis. However, there is no quantitative synthesis of performance of these methods. This systematic review and meta-analysis evaluate the diagnostic performance of AI models for strabismus screening.

**Methods:**

We searched Ovid, Web of Science, PubMed, and Cochrane CENTRAL from inception to May 2025 for studies assessing AI-based strabismus screening. Eligible studies were analyzed using random-effects bivariate models. Subgroup analyses explored the influence of algorithmic architecture (End-to-End vs. Step-by-Step), validation type (internal vs. external), data augmentation, training sample size, and data modality (images, videos, eye-tracking data).

**Results:**

The 24 studies involving at least 8484 patients and 40 394 ocular measurements were included. AI models exhibited strong diagnostic performance, with a pooled sensitivity of 0.94 (95% CI: 0.91–0.97) and specificity of 0.94 (95% CI: 0.91–0.96). End-to-End models (14 studies) had comparable summary sensitivity [0.95 (0.91–0.97) vs. 0.94 (0.85–0.97); *P* = 0.694] and specificity [0.94 (0.91–0.97 vs. 0.93 (0.85–0.97); *P* = 0.627] than Step-by-Step models (10 studies). Subgroup analyses indicated that, for End-to-End models, image-based studies outperformed video-based studies, with higher sensitivity (0.96 vs. 0.85, *P* = 0.04) and specificity (0.95 vs. 0.91, *P* = 0.10). Larger training datasets and data augmentation enhanced performance, though differences were not statistically significant. Most studies demonstrated low risk of bias and applicability concerns across QUADAS-2 domains, except for the index test domain.

**Conclusion:**

AI models exhibit robust performance in strabismus screening, with End-to-End models demonstrating greater consistency. Future research should focus on integrating multimodal data and including diverse populations to enhance the precision and clinical utility of AI-driven strabismus diagnosis.

## Introduction

Strabismus is a common ocular disorder characterized by the misalignment of the eyes^[1]^, preventing simultaneous alignment when focusing on a target. Globally, it affects approximately 1.93% of the population, while in eastern China, the prevalence is notably higher at 5.56% among preschool children aged 48–60 months^[2-4]^. Although it predominantly affects children, strabismus can occur at any age and significantly impacts visual function^[5]^, psychological well-being^[6,7]^, work opportunities^[8]^, and social interactions^[9,10]^, posing a major public health issue. Early diagnosis and timely intervention can greatly improve treatment outcomes. In clinical strabismus examinations, techniques such as the cover test (including cover-uncover and alternate cover methods), Hirschberg test, and alternate prism-cover test (APCT) require substantial training. The present screening methods above are laborious, time-consuming, and necessitate expert clinicians. Even among ophthalmologists, without subspecialty expertise in strabismus or pediatric ophthalmology, proficiency in these skills is often limited. Therefore, there is an urgent need for an intelligent diagnostic system that combines speed, convenience, cost-effectiveness, and high precision to facilitate the referral of potential strabismus patients to subspecialists.

**HIGHLIGHTS**

- First systematic meta-analysis evaluating AI models for strabismus screening across 24 studies.
- AI demonstrated excellent diagnostic accuracy (sensitivity: 0.94, specificity: 0.94).
- Deep learning-based End-to-End models outperformed traditional Step-by-Step methods.
- Image modalities achieved higher sensitivity; eye-tracking modalities provided higher specificity.
- Multimodal data integration and diverse populations are recommended for future AI research.

Recent advances in artificial intelligence (AI) have revolutionized medical diagnostics, particularly in ophthalmology, where AI has shown remarkable success in screening and diagnosing conditions, including keratoconus^[11]^, cataracts^[12]^ and other anterior segment diseases^[13]^, as well as posterior segment diseases such as retinal disorders^[14]^ and optic nerve-related conditions^[15]^. For strabismus, AI-based approaches are emerging as promising tools for automated screening and diagnosis^[16]^. AI algorithms for strabismus are categorized into two types: deep learning-based End-to-End models and traditional machine learning-based Step-by-Step models. End-to-End models, such as convolutional neural networks, automatically derive features from ocular data, offering strong pattern recognition capabilities but requiring significant computational resources and providing limited interpretability^[17]^. Step-by-Step models, such as Support Vector Machines, rely on sequential feature extraction, delivering higher interpretability and lower computational demands, yet they are constrained by feature quality and difficulties with high-dimensional data^[18]^. These systems analyze diverse data modalities, including ocular photographs^[19]^, videos^[20]^, and eye movement data^[21]^, to enhance diagnostic accuracy, reduce healthcare burdens, and minimize misdiagnosis. Despite the growing body of research on AI applications in strabismus screening, there is no quantitative synthesis of their screening performance, including the influence of study design factors such as sample size, algorithm type, and data modality.

This systematic review and meta-analysis aimed to evaluate the performance of AI models designed for strabismus screening. We systematically assessed various study designs, including training sample sizes, different AI algorithms, and diverse data modalities to determine their impact on clinical screening and diagnostic efficacy. The manuscript was thoroughly reviewed following the TITAN Guidelines 2025^[22]^. By synthesizing the available evidence, this study provides insights into the performance of AI-based strabismus screening systems and to identify methodological improvements for future research in this field.

## Methods

This systematic review and meta-analysis were prospectively registered with PROSPERO (CRD42020186641) on 6 April 2025, and the study protocol was uploaded prior to the initiation of data analysis. The reporting of this systematic review adheres to the Preferred Reporting Items for Systematic Reviews and Meta-Analyses (PRISMA)^[23]^ and the Preferred Reporting Items for a Systematic Review and Meta-analysis of Diagnostic Test Accuracy Studies (PRISMA-DTA)^[24]^ guidelines, and the methodological approach aims to meet the standards of the A Measurement Tool to Assess Systematic Reviews (AMSTAR)^[25]^. All stages of the review, including title and abstract screening, full-text screening, data extraction, assessment of reporting guideline adherence, risk of bias, and applicability, were conducted independently by two reviewers (Z.Y.Y. and D.W.W.), and disagreements were resolved through discussion with a third independent reviewer (L.Q.L.).

### Search strategy and selection criteria

We performed a comprehensive search of bibliographic databases, including Ovid, Web of Science, PubMed and Cochrane CENTRAL, to identify studies evaluating the diagnostic accuracy of AI models for strabismus screening. The search spanned from database inception to the final search date, which was updated prior to publication (anticipated in May 2025). The detailed search strategy that combined free texts and MeSH terms relating to the target condition (strabismus) and the index tests (AI screening and diagnosis) are showed in supplementary materials. We also manually searched reference lists and citations of included studies and relevant reviews to identify additional eligible studies. No restrictions were applied regarding publication year, language, or study design during the initial search.

Two reviewers independently screened titles and abstracts using predefined inclusion and exclusion criteria, with citations managed and duplicates removed using EndNote V.X9 (Clarivate Analytics, Pennsylvania, USA). We included all diagnostic accuracy studies, such as clinical trials, cross-sectional studies, prospective and retrospective cohort studies, and case–control studies, that evaluated AI models for screening using ocular data with clinical diagnosis by ophthalmologists as the reference standard. Exclusion criteria included studies focusing on surgical planning, prognostication, or only measuring ocular parameters without diagnostic classification, as well as reviews, case reports, and studies limited to participants with pre-diagnosed strabismus. No restrictions were applied to patient age, gender, ethnicity, geographical location, or sample size.

### Data extraction

Two reviewers independently extracted data using a predefined, standardized data extraction sheet. Study authors were contacted for additional data or clarification if necessary. Extracted data included study characteristics (title, authors, year, country, design), characteristics of sample set (age, inclusion and exclusion criteria, sampling method, sample size), index test details (AI algorithms, number of images for model training/testing, training/validation/testing proportions, ocular data type), reference standard details (diagnostic criteria), and performance metrics (sensitivity, specificity, AUC, and raw data for 2 × 2 contingency tables). Ocular data (images, videos, or eye tracking data) were used as the unit of analysis. Due to the lack of individual participant data, we relied on aggregate data, which may have resulted in overly precise estimates. When multiple AI performance estimates were reported within a study, we only included the best performing AI model based on the highest sensitivity in the meta-analyses as we were interested in study-level outcomes.

### Data synthesis and analysis

We constructed 2 × 2 contingency tables to calculate sensitivity and specificity, presenting point estimates with 95% confidence intervals (CIs) in paired forest plots for each study. Summary receiver operating characteristic (SROC) plots were generated, with 95% confidence and prediction regions surrounding the point estimates. We did not use the I^2^ statistic commonly applied in intervention meta-analyses to assess heterogeneity. This decision was based on several limitations of I^2^ in the diagnostic test accuracy (DTA) reviews: it is unable to account for heterogeneity caused by threshold effects between sensitivity and specificity, and does not consider the mean–variance relationship of sensitivity and specificity, which may result in biased estimates. We anticipated heterogeneity among AI models and algorithms but accepted it, as our review aimed to evaluate the diagnostic performance of any AI model for strabismus screening. Given the potential biases associated with I^2^ in DTA reviews, we primarily relied on SROC plots to visually assess heterogeneity, as recommended by the Cochrane Handbook for Systematic Reviews of Diagnostic Test Accuracy^[26]^.

Given anticipated heterogeneity in AI algorithms used across studies, we used random-effects models for all meta-analyses. Sensitivity and specificity were jointly synthesized using a bivariate model, implemented with the midas and meqrlogit commands in Stata 18.

Bivariate meta-regression was used to compare the diagnostic performance of End-to-End and Step-by-Step models, with absolute differences in sensitivity and specificity computed post-estimation using the nlcom command and *P* values derived from Wald tests. Given the substantial differences in datasets, model architecture, and learning approaches between End-to-End and Step-by-Step models, subgroup analyses were conducted separately for each model type to investigate sources of within-group heterogeneity. For End-to-End models, covariates included validation type (internal or external), data augmentation (yes or no), training set sample size (grouped by a median of 4000) and data type (images, videos or eye tracking data). For Step-by-Step models, covariates included data type (images, videos or eye tracking data).

### Quality assessment

The methodological quality of included studies was evaluated using the Quality Assessment of Diagnostic Accuracy Studies-2 (QUADAS-2) tool, assessing risk of bias and applicability concerns across four domains: patient selection, index test, reference standard, and flow and timing^[27]^.

### Publication bias

We assessed publication bias using Deek’s funnel plot asymmetry test, with *P* <0.05 indicating significant publication bias. To minimize publication bias, we included gray literature and manually searched reference lists of included studies and relevant reviews.

### Declaration of artificial intelligence use

According to TITAN Guidelines 2025^[22]^, no artificial intelligence (AI) tools were used in the research or manuscript development of this study. All research design, data analysis, result interpretation, and manuscript writing were conducted independently by the research team to ensure the integrity and transparency of the study.

## Results

**Figure 1.**
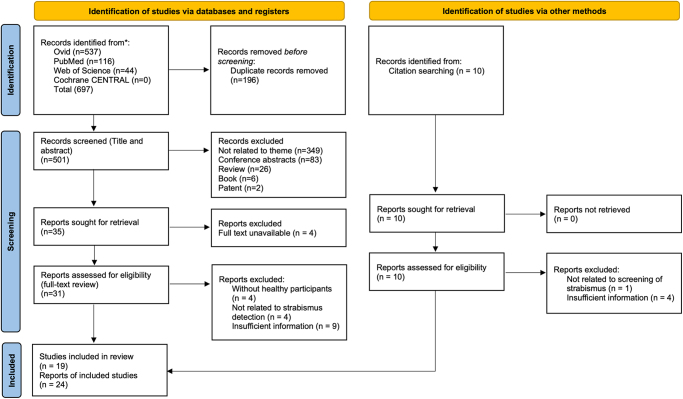
PRISMA 2020 flow diagram for Systematic Reviews and Meta-Analyses flowchart shows studies selected for review. CENTRAL = Central Register of Controlled Trials.

We identified 697 peer-reviewed studies, of which 196 were duplicates. A further 10 studies were identified through citation searching. After full-text screening, 24 studies in 19 publications met our inclusion criteria (Fig. 1). Five publications (Chen *et al*^[20]^., Zheng *et al*^[28]^., Mao *et al*^[29]^., Wu *et al*^[19]^. and Kurdthongmee *et al*^[30]^.) reports two studies using different sample sets in one publication, respectively.

### Characteristics of eligible studies

The 24 studies, published before May 2025, included at least 8484 patients and 40 394 ocular measurements.^[17–21,28–41]^ Nine studies^[19,28–30,32,33,35,36,40]^ reported only ocular measurements, not participant numbers. Studies were conducted in four countries, with China being the most common (66.7%). Key characteristics of the included studies are summarized in Table 1.

**Table 1:** Summary of key characteristics of included studies

Authors (year)	Country	Age (year range)	Inclusion criteria	Exclusion criteria	No. of subjects	Data type	No. of data	Target condition
Long *et al* (2019)	China	0–3	Visual impairment (including strabismus)	Brain or mental illnesses; other known illnesses that could affect their behavioral patterns	4196	Videos	4196	Strabismus versus other 10 behavior phenotypes
Chen *et al* (2023)	China	0–4	Visual impairment (including strabismus)	Central nervous system diseases, mental illnesses or other known illnesses that could affect their behavioral patterns in the absence of ocular manifestations; Receiving interventions and treatments in last one month; uncooperative for examinations or data collection	3652	Videos	3652	Strabismus from the other categories (aphakia, congenital glaucoma, nonimpairment, congenital ptosis)
Valente *et al* (2017)	Brazil	NR	Exotropia	NR	7	Eye tracking data	15	Exotropia versus normal
Chen *et al* (2018)	China	25–63	Recessive, intermittent, and manifest strabismus	NR	42	Eye tracking data	42	Strabismus versus normal
Ma *et al* (2020)	China	8–10	Strabismus, myopia and anisometropia	NR	100	Images	100	Strabismus versus no strabismus (normal, myopia, anisometropia)
N. Khumdat *et al* (2013)	Thailand	11–17	Strabismus	NR	103	Images	206	Strabismus versus normal
Almeida *et al* (2012)	Brazil	NR	Strabismus	Horizontal and/or vertical yaw above 15△; opacity or any other alteration of the cornea and/or eyelid that compromises the observation of luminous reflections in both corneas; irregularity on the limbus contour; nystagmus; iris or pupil alterations; alterations in the size of one of the eyes	45	Images	45	Strabismus versus normal
Simoes *et al* (2019)	Brazil	NR	Exotropia, esotropia, hypertropia and hypotropia	NR	45	Images	225	Strabismus versus normal
Mesquita *et al* (2021)	Brazil	5–15	Exotropia, esotropia, hypertropia and hypotropia	NR	224	Images	224	Strabismus versus normal
Zheng *et al* (2021)	China	NR	Horizontal strabismus	Children with restrictive strabismus, sensory strabismus, paralytic strabismus, myasthenia gravis, nystagmus, and Duane syndrome	NR	Images	7303	Horizontal strabismus versus normal
Mao *et al* (2021)	China	1–65	Horizontal strabismus	Photos with blurred reflex points; reflex points outside the edge of the cornea; presence of vertical strabismus; absence of full deviation for intermittent exotropia	NR	Images	5797	Strabismus versus normal
Wu *et al* (2024)	China	1–74	Exotropia, esotropia and vertical deviation	Photographs featuring extraneous noise points, or reflex points obscured by occurrences of blepharoptosis, or the absence of reflex points; photographs depicting indistinct or defocused reflex points	NR	Images	6194	Strabismus versus normal
Shu *et al* (2024)	China	0–18	Strabismus	Ocular conditions affecting image capture; severe facial abnormalities; history of strabismus or ptosis reconstructive surgery; psychological or psychiatric issues	NR	Images	473	Strabismus versus myopia and ptosis
Kurdthongmee *et al* (2024)	Thailand	NR	Strabismus	NR	NR	Images & videos	1236 images, 12 videos	Strabismus versus normal
Yarkheir *et al* (2025)	Iran	NR	Exotropia and esotropia	Low-quality, inaccurate photos or wearing masks; participants with both vertical and horizontal abnormalities	NR	Images	4879	Horizontal strabismus versus normal
Zhao *et al* (2025)	China	8–50	Exotropia	Other serious ocular conditions besides exotropia	70	Eye tracking data	70	Exotropia versus normal
Jabbar *et al* (2024)	China	NR	NR	NR	NR	Eye tracking data	NR	Exotropia, esotropia, hypotropia and hypertropia versus normal
Lu *et al* (2018)	China	NR	NR	NR	NR	Images	5685	Strabismus versus normal
Cheng *et al* (2021)	China	NR	NR	NR	NR	Images	40	Strabismus versus normal

Authors (year)	Task	Reference standard^a^	Type of internal validation	External validation	Training/validation/testing ratio^b^	Data augmentation	AI algorithm	Algorithmic decision Mechanism	Output
Long *et al* (2019)	Diagnosis	Expert consensus	Stratified random sampling	No	80:20	No	Temporal segment networks	End-to-End	AUC = 0.902 (0.876–0.924)
									Sensitivity = 94.2 (92.0–96.0)
									Specificity = 84.3 (71.4–93.0)
Chen *et al* (2023)	Diagnosis	Expert consensus	Stratified random sampling	Yes	80:20	No	EfficientNet-B4	End-to-End	AUC = 0.918 (0.875–0.961)
	Screening								AUC = 0.973 (0.956–0.991)
									Accuracy = 0.885 (0.806–0.935)
									Specificity = 0.896 (0.778–0.955)
									Sensitivity = 0.875 (0.753–0.941)
Valente *et al* (2017)	Diagnosis	Expert consensus	NR	NR	NR	No	Image processing	Step-by-Step	Sensitivity = 0.800
									Specificity = 1.000
									Accuracy = 0.933
Chen *et al* (2018)	Screening	Expert consensus	“Leave-one-out” method	NR	41:1	No	VGG-S, AlexNet, VGG-F, VGG-M, VGG-16, VGG-17	End-to-End	VGG-S:
									Accuracy = 0.952
									Specificity = 0.960
									Sensitivity = 0.941
Ma *et al* (2020)	Screening	Expert consensus	NR	NR	NR	No	Image processing	Step-by-Step	Accuracy = 0.940
									Specificity = 0.980
									Sensitivity = 0.800
N. Khumdat *et al* (2013)	Screening	Expert consensus	NR	NR	NR	No	Image processing	Step-by-Step	Accuracy = 0.942
									Specificity = 0.731
									Sensitivity = 0.972
Almeida *et al* (2012)	Screening	Expert consensus	NR	NR	NR	No	Image processing, support vector machines	Step-by-Step	Accuracy = 0.940
									Specificity = 0.913
									Sensitivity = 1.000
Simoes *et al* (2019)	Diagnosis	Expert consensus	NR	NR	NR	Yes	U-Net, ResNet	Step-by-Step	Accuracy = 0.966
									Specificity = 1.000
									Sensitivity = 0.958
Mesquita *et al* (2021)	Screening	Expert consensus	NR	NR	NR	No	Image processing	Step-by-Step	Accuracy = 0.845
									Specificity = 0.844
									Sensitivity = 0.895
									Kappa coefficient = 0.430
Zheng *et al* (2021)	Screening	Expert consensus	Random sampling	Yes	80:20	Yes	Inception-V3, VGG16, Xception	End-to-End	Internal validation:
									AUC = 0.993 (0.989–1.000) (inception-V3), 0.993 (0.989–1.000) (VGG16), 0.991 (0.986–1.000) (Xception)
									External validation (inception-V3):
									Accuracy = 0.968 (0.947–0.989)
									Specificity = 0.993 (0.983–1.000)
									Sensitivity = 0.940 (0.919–0.968)
Mao *et al* (2021)	Screening	Expert consensus	Random sampling	Yes	70:15:15	Yes	InceptionResNetV2	End-to-End	For test set (870 images):
									AUC = 0.998 (0.993–1.000)
									Accuracy = 0.990 (0.980–0.995)
									Specificity = 0.983 (0.946–0.995)
									Sensitivity = 0.991 (0.980–0.997)
									For external validation:
									AUC = 0.980 (0.963–0.997)
									Accuracy = 0.972 (0.955–0.983)
									Specificity = 0.857 (0.741–0.929)
									Sensitivity = 0.986 (0.971–0.994)
Wu *et al* (2024)	Screening	Expert consensus	Random sampling	Yes	80:20	Yes	VIT_16_224	End-to-End	Internal validation:
									Accuracy = 0.980 (0.962–0.993)
									Specificity = 0.979 (0.961–0.991)
									Sensitivity = 0.958 (0.925–0.988)
									AUC = 0.994 (0.990–0.999)
									External validation:
									Accuracy = 0.967
									Specificity = 0.970
									Sensitivity = 0.960
									AUC = 0.993
Shu *et al* (2024)	Screening	Expert consensus	NR	NR	NR	Yes	ConvNeXt	End-to-End	AUC = 0.83 (0.82–0.85)
									Accuracy = 0.80 (0.79–0.82)
									Sensitivity = 0.73(0.70–0.77)
									Specificity = 0.85 (0.84–0.86)
Kurdthongmee *et al* (2024)	Screening	NR	NR	Yes	NR	No	Xception	Step-by-Step	Percent correctness of images = 96.67% (normal), 100% (strabismus)
									Percent correctness of videos = 100% (normal), 83.33% (strabismus)
Yarkheir *et al* (2025)	Screening	Expert consensus	Random sampling	No	80:20	Yes	ResNet101	End-to-End	Accuracy = 0.8638
									F1 = 0.8639
									Recall = 0.8638
									Precision = 0.8641
	Diagnosis		NR	NR	NR				Accuracy = 0.927083
									F1 = 0.910853
									Recall = 0.927083
									Precision = 0.895181
Zhao *et al* (2025)	Screening	Expert consensus	“Leave-one-out” method	No	69:1	No	The random forest	Step-by-Step	Accuracy = 0.971
									Precision = 0.971
Jabbar *et al* (2024)	Diagnosis	NR	NR	NR	NR	Yes	FedCNN	End-to-End	Accuracy = 0.952
									F1 = 0.93
									Recall = 0.92
									Precision = 0.94
Lu *et al* (2018)	Screening	NR	NR	NR	60:40	No	RF-CNN	End-to-End	Sensitivity = 0.9330
									Specificity = 0.9617
									Accuracy = 0.9389
									AUC = 0.9865
Cheng *et al* (2021)	Screening	Expert consensus	NR	NR	NR	No	Image processing	Step-by-Step	Sensitivity = 0.833
									Specificity = 0.765

AUC = area under the ROC curve; ET, esotropia; HoT, hypotropia; HT, hypertropia; NR, not report; XT, exotropia.

^a^ Expert consensus = diagnosis of strabismus is made by one or more ophthalmologists.

^b^ Some studies only have training and validation dataset.

All 24 studies were cross-sectional studies, and ocular data were obtained from eligible volunteers, local screening programs or hospitals. Thirteen (54.2%) studies described the demographic characteristics of their study population: seven (29.2%) included only subjects under 18 years old^[17,18,20,34,36,38]^, one (4.2%) included only adults^[21]^, and five (20.8%) had no age restrictions^[19,29,41]^. Twenty studies (83.3%) focused on strabismus screening (distinguishing strabismus from healthy subjects or other ocular diseases), with 16 comparing strabismus to healthy eyes^[19,21,28–32,34,35,38,40,41]^ and four on strabismus versus healthy eyes and/or other ocular disease^[17,18,20,36]^. Four studies (16.7%) diagnosed specific strabismus types, including esotropia, hypotropia, hypertropia, exotropia^[20,33,37,39]^. To ensure analytical consistency, we reclassified the results of these studies into a binary classification task, distinguishing strabismus from non-strabismus. Specifically, all strabismus subtypes were categorized as positive cases, while non-strabismus cases (including healthy eyes or other non-strabismus ocular conditions) were categorized as negative cases, based on the diagnostic outcomes reported in each study.

Eight studies excluded participants with other conditions that could affect ocular manifestations^[17,20,28,31,36,41]^, five excluded low-quality images^[19,29,40]^, and eleven did not report exclusion criteria^[18,21,30,32–35,37–39]^. Most studies (83.3%) used expert consensus for the reference standard, with 14 reporting use of the alternate prism-cover test (APCT), the cover test or Hirschberg test^[17–20,28,29,31,32,36,40]^. Four studies did not specify reference standard methods^[30,33,35]^.Sixteen studies used digital cameras or smartphone to acquire ocular images^[18,19,28–32,34–38,40]^, four used eye tracking data^[21,33,39,41]^ and four used videos^[17,20,30]^.

Fourteen (58.3%) studies used End-to-End model^[17,19,20,28,29,35,36,40]^, employing 10 CNN-based architectures including Inception architecture, VGGNet, EfficientNet-B4, VIT_16_224, U-Net, ResNet, ConvNeXt and FedCNN. The remaining 10 studies used Step-by-Step models^[18,21,30–34,37–39,41]^. Ten (41.7%) studies applied data augmentation^[19,28,29,33,36,37,40]^, and five (20.9%) used external validation^[19,20,28–30]^. Seven (29.2%) studies used random split sample validation as a method of internal validation^[19,28,29,40]^, three used stratified split sampling^[17,20]^, and two used the leave-one-out method^[21,41]^. Some studies only have training and validation datasets. Table 1 shows the detailed characteristics of included studies.

### Quality assessment

Supplementary Digital Content Table 1 (available at: http://links.lww.com/JS9/E762) and Supplementary Digital Content Figure 1 (available at: http://links.lww.com/JS9/E762) summarize the risk of bias assessment. Regarding patient selection, risk of bias was low in 15 studies, unclear in 9 studies^[21,30,33–35,37,39,41]^ due to the insufficient information describing source/process of patient selection. With respect of index test, neural networks’ screening was based on algorithms without knowledge of the doctors’ diagnosis in all studies, though 19 studies^[17,19–21,28–30,32,33,35,36,38,40,41]^ were judged as high risk of bias owing to the lack of pre-specified threshold. As for reference standard, risk of bias was low in 20 studies and high in 4 studies^[30,33,35]^ due to unclear methods. Nine studies^[29–31,33,35,40,41]^ had an unclear risk in flow and timing domain due to the potential inconsistency of reference standard used or incomplete data inclusion. For applicability, most studies had low concern regarding patient selection (17 studies) and reference standard (20 studies) but high concern in the index test domain (19 studies) due to potential overestimation of the diagnostic performance of AI because of the lack of threshold pre-specification.

### Meta-analysis

**Figure 2.**
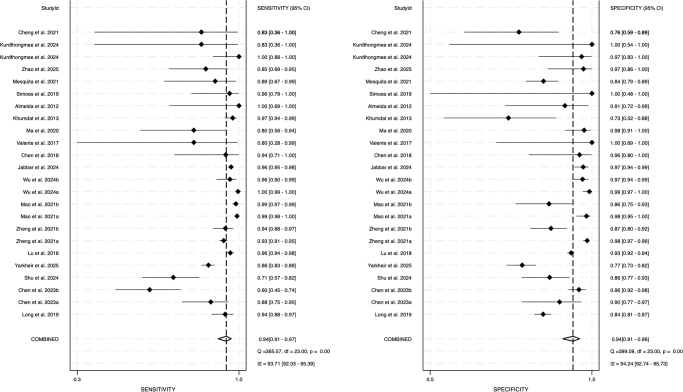
Forest plots demonstrating the sensitivity and specificity of each included studies.

**Figure 3.**
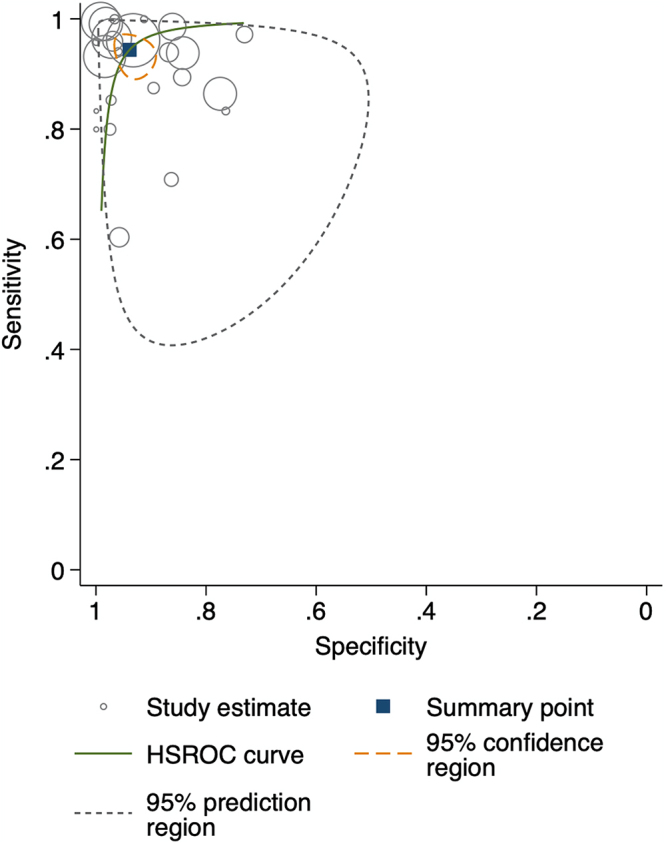
Summary receiver operating characteristic (SROC) plots for of eligible studies (24 studies, 11 174 images).

**Figure 4.**
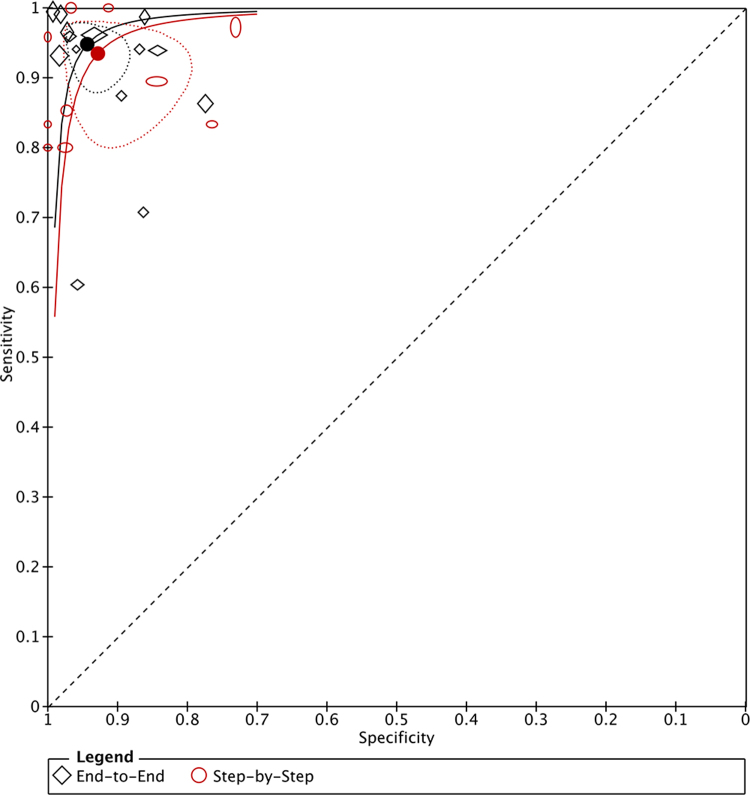
Summary receiver operating characteristic (SROC) plot of accuracy of End-to-End models (14 studies, 10 384 images) versus Step-by-Step models (10 studies, 790 images). The markers for each test on the SROC plots represent pairs of sensitivity and specificity from the included studies. The hollow symbols are the study points for each index test. The size of each marker was scaled according to the precision of sensitivity and specificity in the study. The solid circles are the summary points representing the summary sensitivities and specificities. Each summary point is surrounded by a dotted line representing the 95% confidence region (illustrating the uncertainty around the estimates of sensitivity and specificity) and a dashed line representing the 95% prediction region (the region within which one is 95% certain the results of a new study will lie).

Figure 2 shows the paired forest plot of sensitivity and specificity with corresponding 95% CIs for each study. Eligible studies were further combined using the hierarchical summary receiver operating characteristic (HSROC) model, and the SROC curve is shown in Fig. 3 with the 95% confidence region and 95% prediction region. We calculated the following summarized estimates using the HSROC model: sensitivity 0.94 (95% CI: 0.91–0.97), specificity 0.94 (95% CI: 0.91–0.96) (Table 2). Comparing End-to-End (14 studies, 10 384 ocular measurements) and Step-by-Step models (10 studies, 790 ocular measurements), End-to-End models had higher sensitivity [0.95 (0.91–0.97) vs. 0.94 (0.85–0.97), *P* = 0.694] and specificity [0.94 (0.91–0.97 vs. 0.93 (0.85–0.97), *P* = 0.627] than Step-by-Step models (Table 2 and Fig. 4). Absolute differences were 1.3% (95% CI: −5.3 to 8.0) for sensitivity and 1.5% (95% CI: −4.6 to 7.7) for specificity. Although not statistically significant, End-to-End models had smaller 95% confidence regions, indicating more stable performance. Coupled forest plots for both models are shown in Supplementary Digital Content Figure 2 (available at: http://links.lww.com/JS9/E762).

**Table 2:** Overview of meta-analytic results of the performance of artificial intelligence models for strabismus

Model (N = # studies | n = # data)	Sensitivity (95% CI)		*P* value	Specificity (95% CI)		*P* value
1. AI performance						
Overall (N = 24 | n = 11 174)	0.94	(0.91–0.97)	–	0.94	(0.91–0.97)	–
2. End-to-End versus Step-by-Step^a^						
End-to-End (N = 14 | n = 10 384)	0.95	(0.91–0.97)	0.694	0.94	(0.91–0.97)	0.627
Step-by-Step (N = 10 | n = 790)	0.94	(0.85–0.97)	–	0.93	(0.85–0.97)	–

^a^ Statistical comparison made between End-to-End and Step-by-Step groups using bivariate meta-regression with Wald tests. *P* value of <0.05 is considered statistically significant.

### Subgroup analysis

**Figure 5.**
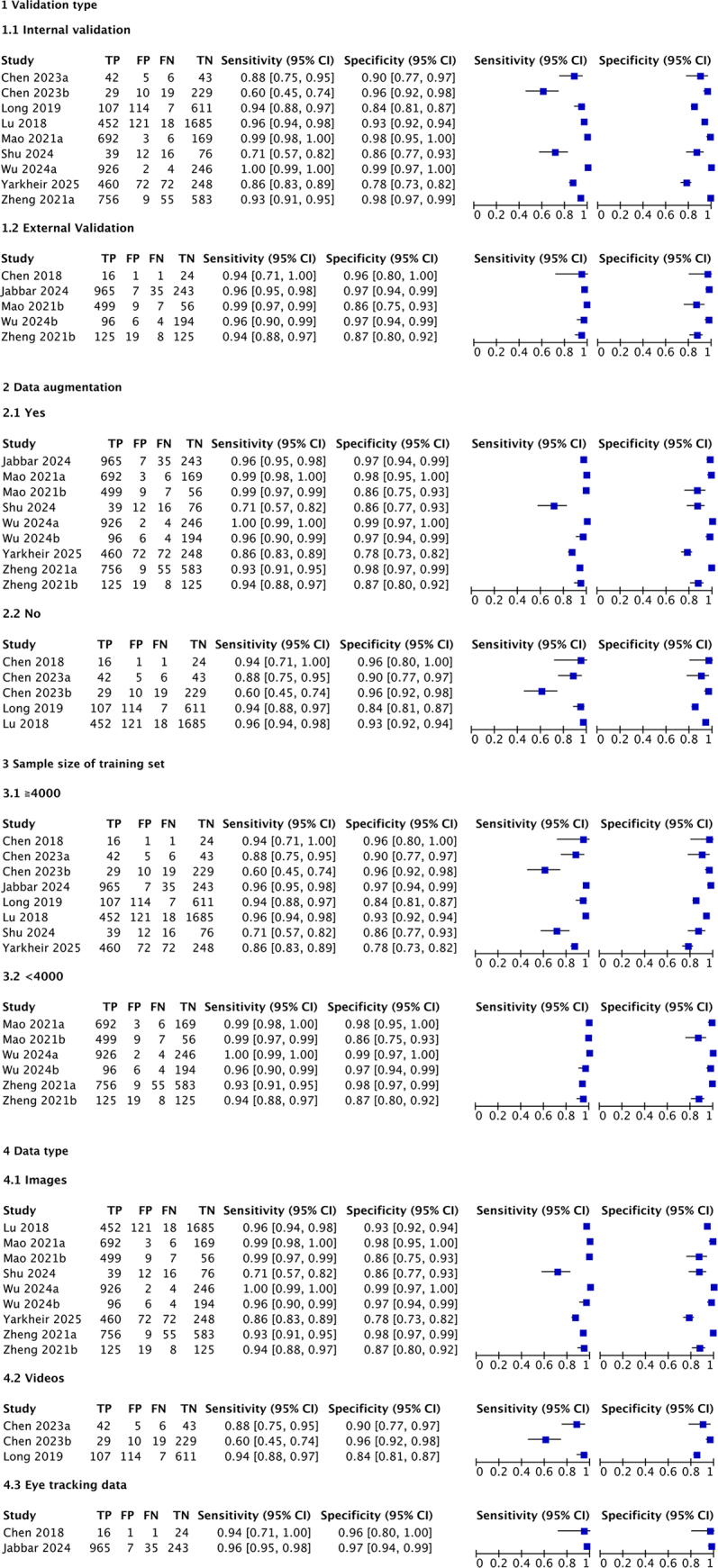
Coupled forest plots for End-to-End algorithms (N = 14) in four subgroups separated by validation type, data augmentation, sample size of training set and data type.

**Figure 6.**
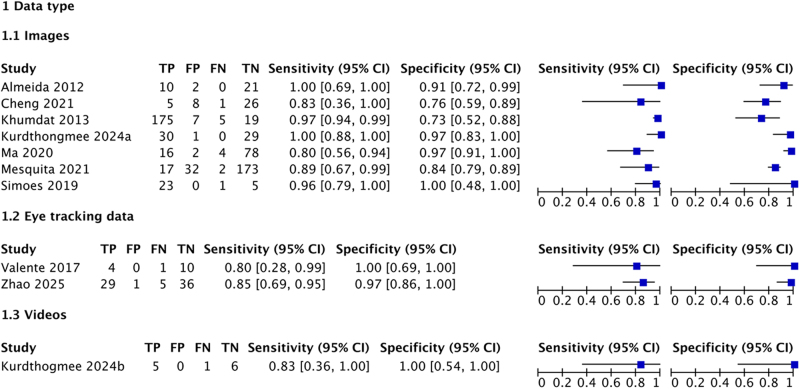
Coupled forest plots for Step-by-Step algorithms (N = 12) in subgroup separated by data type. (Please note that for optimal clarity, reviewers are kindly requested to click the “click here to access/download” link in the top-right corner of each figure to view the high-resolution original image.).

Subgroup analyses were conducted separately for End-to-End and Step-by-Step models to explore sources of heterogeneity, as described in the Methods. For End-to-End models, image-based studies outperformed video-based studies, with pooled sensitivity and specificity of 0.96 (95% CI: 0.93–0.99) and 0.95 (95% CI: 0.91–0.98) for images, compared to 0.85 (95% CI: 0.66–1.00, *P* = 0.04) and 0.91 (95% CI: 0.81–1.00, *P* = 0.10) for videos. Other covariates in End-to-End models, including validation type (internal or external), data augmentation (yes or no), and training set sample size, showed no significant differences in random-effects bivariate logistic models. For Step-by-Step models, no association was found between model performance and data type. Detailed subgroup analysis results are presented in Table 3, with coupled forest plots for each subgroup in Figs. 5 and 6.

**Table 3:** Subgroup analyses for the performance of automatic detection of strabismus using End-to-End models and Step-by-Step models

Subgroup variables	Studies	Sensitivity (95% CI)	*P* value	Specificity (95% CI)	*P* value
End-to-End	14				
Internal/external validation					
Internal validation	9	0.93 (0.88–0.99)	0.06	0.94 (0.91–0.98)	0.10
External validation	5	0.97 (0.93–1.00)	-	0.94 (0.89–1.00)	-
Data augmentation					
Yes	9	0.96 (0.93–0.99)	0.92	0.95 (0.92–0.98)	0.21
No	5	0.91 (0.81–1.00)	-	0.93 (0.86–0.99)	-
Sample size of training set^a^					
≧4000	8	0.98 (0.96–0.99)	0.80	0.97 (0.95–0.99)	0.27
<4000	6	0.87 (0.78–0.96)	-	0.89 (0.82–0.96)	-
Data type					
Images^b^	9	0.96 (0.93–0.99)	0.04^b^	0.95 (0.91–0.98)	0.10
Videos^b^	3	0.85 (0.66–1.00)	-	0.91 (0.81–1.00)	-
Eye tracking data	2	0.96 (0.89–1.00)	-	0.97 (0.92–1.00)	-
Step-by-Step	10				
Data type					
Images	7	0.95 (0.92–0.98)	0.92	0.89 (0.82–0.96)	0.18
Eye tracking data & videos	3	0.84 (0.71–0.97)	-	0.99 (0.95–1.00)	-

a 4000 was selected as the grouping threshold, as it is the median of the training set sample size.

b The reported *P* values indicate the statistical significance of sensitivity (*P* = 0.04) and specificity (*P* = 0.10) differences between the images vs. videos subgroups. No significant differences were found in other subgroup comparisons.

### Publication bias

Publication bias was assessed using regression analysis of funnel plot asymmetry (Supplementary Digital Content Figure 3, available at: http://links.lww.com/JS9/E762). The slope coefficient was −17.3 (95% CI: −44.4 to 9.8; *P* = 0.20), indicating a low risk of publication bias.

## Discussion

In this systematic review and meta-analysis, we evaluated the diagnostic performance of AI models for strabismus screening. To investigate the impact of study design on model performance, we conducted subgroup analyses, assessing the effects of algorithmic architecture, validation type, data augmentation, training set sample size, and data modality. Random-effects bivariate models were employed to synthesize sensitivity and specificity, with summary receiver operating characteristic (SROC) plots used to visually assess heterogeneity, providing a comprehensive evaluation of AI model performance in strabismus screening.

AI models for strabismus screening exhibited robust diagnostic performance, with an overall sensitivity of 0.94 (95% CI: 0.91–0.97) and specificity of 0.94 (95% CI: 0.91–0.96), suggesting substantial clinical potential. Studies were categorized by algorithmic architecture into End-to-End and Step-by-Step models. End-to-End models, driven by deep learning techniques such as convolutional neural networks, autonomously extract features from diverse ocular data (images, videos, and eye-tracking data), facilitating superior pattern recognition and generalizability despite high computational demands and limited interpretability^[42]^. In contrast, Step-by-Step models, based on traditional machine learning, rely on sequential preprocessing and feature extraction using methods like Support Vector Machines^[43,44]^. These models offer greater interpretability and lower computational requirements but are constrained by their dependence on feature quality, often struggling with high-dimensional data. End-to-End models outperformed Step-by-Step models, demonstrating higher sensitivity [0.95 (95% CI: 0.91–0.97) vs. 0.94 (95% CI: 0.85–0.97); *P* = 0.694] and specificity [0.94 (95% CI: 0.91–0.97) vs. 0.93 (95% CI: 0.85–0.97); *P* = 0.627], with absolute differences of 1.3% (95% CI: −5.3 to 8.0) for sensitivity and 1.5% (95% CI: −4.6 to 7.7) for specificity. Although not statistically significant, End-to-End models displayed narrower 95% confidence regions, indicating more consistent diagnostic performance. Notably, End-to-End models achieved comparable sensitivity and specificity across internal and external validation sets, whereas Step-by-Step models often employed pre-trained models, such as facial detection models, to extract key eye regions from facial images, calculating coordinates of pupil centers and corneal light reflex points for comparison against predetermined thresholds, thereby identifying the presence and subtype of strabismus, typically on a single validation dataset without distinguishing between internal and external validation. Although this approach enhances interpretability, it relies on thresholds derived from limited datasets, introducing potential selection bias and reducing generalizability.

Further subgroup analyses were conducted on the 14 End-to-End and 10 Step-by-Step studies to explore factors influencing diagnostic performance. For End-to-End models, we assessed the impact of training dataset size by dividing studies based on the median sample size of 4000 into larger (≥4000, 8 studies) and smaller (<4000, 6 studies) groups. Studies with larger training samples demonstrated higher sensitivity [0.98 (95% CI: 0.96–0.99) vs. 0.87 (95% CI: 0.78–0.96); *P* = 0.80] and specificity [0.97 (95% CI: 0.95–0.99) vs. 0.89 (95% CI: 0.82–0.96); *P* = 0.27], with narrower 95% confidence regions, indicating more consistent diagnostic performance, though the improvement was not statistically significant. Similarly, data augmentation in End-to-End models (9 studies) enhanced sensitivity [0.96 (95% CI: 0.93–0.99) vs. 0.91 (95% CI: 0.81–1.00); *P* = 0.92] and specificity [0.95 (95% CI: 0.92–0.98) vs. 0.93 (95% CI: 0.86–0.99); *P* = 0.21] compared to studies without augmentation (5 studies), but the gains were also non-significant. These findings suggest a diminishing marginal effect of increasing training sample size, indicating that while continuing to expand dataset collection, future research should prioritize alternative strategies, such as refining algorithms, to further enhance model performance. External validation (5 studies) exhibited marginally higher sensitivity [0.97 (95% CI: 0.93–1.00) vs. 0.93 (95% CI: 0.88–0.99); *P* = 0.06] and comparable specificity [0.94 (95% CI: 0.89–1.00) vs. 0.94 (95% CI: 0.91–0.98); *P* = 0.10] relative to internal validation (9 studies), with no statistically significant differences. As most studies were single-center, potential similarities may exist between internal and external validation datasets. Multicenter datasets should be utilized to enhance model generalizability. Additionally, data modality was another key focus, as we evaluated its impact on diagnostic performance across images (9 studies), videos (3 studies), and eye-tracking data (2 studies). Video-based studies exhibited significantly lower sensitivity (0.85 vs. 0.96 for both images and eye-tracking data; *P* = 0.04), likely because they were designed to screen for multiple pediatric eye conditions, including strabismus, rather than focusing solely on strabismus, with the complexity of feature extraction from video data posing additional challenges. Image-based studies demonstrated the highest sensitivity (0.96, 95% CI: 0.93–0.99) with narrower 95% confidence intervals, suggesting that AI-driven screening via photo uploads from smart devices is a feasible approach. Furthermore, eye-tracking data yielded the highest specificity (0.95, 95% CI: 0.91–0.98), possibly because, unlike image- and video-based studies, which may misclassify normal cases as strabismus due to large kappa angles, eye-tracking data assesses binocular visual function through tasks such as saccades and tracking, minimizing interference from appearance-based false positives like large kappa angles. A similar trend was observed in the 10 Step-by-Step studies, where image-based studies (7 studies) showed higher sensitivity (0.95, 95% CI: 0.92–0.98) with narrower confidence intervals but lower specificity (0.89, 95% CI: 0.82–0.96), while studies using eye-tracking data and videos (3 studies) exhibited higher specificity (0.99, 95% CI: 0.95–1.00) with narrower confidence intervals but lower sensitivity (0.84, 95% CI: 0.71–0.97).

According to the American Academy of Ophthalmology (AAO) Preferred Practice Pattern^[45,46]^, strabismus treatment varies by subtype, including targeted surgical or non-surgical interventions such as observation, refractive error correction, vision therapy, or botulinum toxin injections. Accurate diagnosis of strabismus subtypes is therefore essential for effective treatment planning. Despite the impressive performance of AI models in strabismus screening across various study designs, current research primarily focuses on screening tasks, lacking the ability to differentiate strabismus subtypes. Our systematic review reveals that most studies excluded complex strabismus types, such as vertical strabismus, intermittent exotropia, and nystagmus, and predominantly involved pediatric populations. In real-world settings, however, patients of all ages and strabismus subtypes require timely and accurate diagnosis, highlighting the limited generalizability and practical utility of these AI models in diverse scenarios. Additionally, existing studies rely on single-modality data (e.g., images, videos, eye-tracking), with our subgroup analysis indicating that images offer higher sensitivity for screening, eye-tracking data provide greater specificity for identifying normal cases, and videos hold potential for diagnosing specific subtypes like intermittent exotropia by capturing critical frames – yet video-based AI models remain underexplored. In contrast, clinicians integrate multimodal, multi-timepoint data to enhance diagnostic accuracy, underscoring the challenge and opportunity of relying on single data sources. Future research should prioritize experimental designs that include patients of all ages and strabismus subtypes, despite challenges posed by diverse image representations (e.g., palpebral fissure size, eye color, skin tone), which may lead models to learn irrelevant features. Overcoming these challenges is critical for improving clinical utility. Moreover, integrating binocular vision assessment through virtual reality (VR) and biosensors offers promising directions. VR can simulate complex visual environments to capture real-time eye movements and responses^[47–50]^, while biosensors monitor physiological signals (e.g., pupil response, intraocular pressure), providing precise data on binocular coordination and visual fatigue. Combining these technologies with multimodal data – such as eye movement videos, ocular position photos, color fundus photography, orbital MRI, and electronic medical records – can create comprehensive visual function profiles, enabling more accurate diagnosis and tailored treatment strategies for strabismus.

Several limitations are recognized in this systematic review. First, it is difficult to assess the impact of using public databases and the lack of demographic data, as published studies rarely disclose detailed information about the databases used. Additionally, the limited number of studies included in certain subgroups may reduce the robustness of the results, as individual studies could have a disproportionate influence and compromise the precision of the estimates. Second, over half of the studies exhibited high or unclear risk of bias, particularly in the index test domain. For most End-to-End studies, optimal sensitivity and specificity were achieved through parameter optimization rather than identifying an ideal cut-off point or threshold, making it impossible to extract and compare thresholds in the conventional sense. The absence of prespecified thresholds may overestimate model performance via optimization, potentially limiting clinical generalizability. Furthermore, current AI models still exhibit a “black-box” nature – the lack of explainability at the core logic of modern devices that use AI/ML – which to some extent undermines clinicians’ trust in AI^[51]^. Visualization techniques such as Grad-CAM have been developed to enhance the transparency and interpretability of AI models. We did not include studies that performed multimodal analysis, though only one study was identified^[19]^. Third, the conversion of studies diagnosing specific strabismus types into a binary classification task, necessitated by the limited number of such studies, may mask variations in diagnostic complexity and AI model performance across strabismus subtypes, such as esotropia versus exotropia. Future research should investigate AI methods tailored to strabismus subtype diagnosis to assess their efficacy across diverse subtypes.

In conclusion, this systematic review and meta-analysis systematically evaluated AI models in strabismus screening, revealing key insights across training sample size, algorithmic approaches (End-to-End vs. Step-by-Step), and data modalities (images, videos, eye-tracking data). Distinct patterns emerged in clinical screening and diagnostic performance, with End-to-End models demonstrating superior sensitivity, specificity, and consistency, while a diminishing marginal effect of increasing sample size underscored the need for strategies like algorithm refinement. Image-based studies excelled in sensitivity, eye-tracking data in specificity, and videos showed potential for subtype diagnosis. However, the reviewed AI studies often relied on single-modality data, excluded complex subtypes, and focused on pediatric populations, limiting their generalizability in diverse clinical settings. By addressing these gaps through inclusive, multimodal approaches and technologies like virtual reality and biosensors, this meta-analysis offers methodological insights to guide future research in enhancing the precision, generalizability, and clinical utility of AI-driven strabismus diagnosis.

## Data Availability

This study is a systematic review and meta-analysis. All data used were derived from previously published studies and are publicly accessible. Data extraction sheets and statistical analysis code used in this study are available from the corresponding author upon reasonable request.

## Research registration unique identifying number (UIN)

This systematic review and meta-analysis has been registered with PROSPERO under registration number: CRD42020186641.
